# P03.08. The case for a well-being program for residents in training: preliminary findings

**DOI:** 10.1186/1472-6882-12-S1-P261

**Published:** 2012-06-12

**Authors:** H Saadat, D Snow, S Ottenheimer, F Dai, Z Kain

**Affiliations:** 1 Yale University, Woodbridge, USA; 2 University of Irvine, Irvine, USA

## Purpose

This study evaluates the effects of a comprehensive preventive intervention with anesthesia residents at all levels of training at Yale University.

## Methods

This randomized controlled trial was undertaken with 60 anesthesiology residents at a large teaching hospital in an urban area. The study had three groups: 1) wellness intervention group, 2) no-treatment control group with release time (NTC-RT), and 3) no-treatment control group with routine duties (NTC-RD). Residents in the wellness group were given release time in order to participate in a wellness intervention, Coping with Work and Family Stress, which consisted of 16, 1.5-hour weekly sessions. Residents in the NTC-RT group were given the same amount of release time each week and chose activities such as reading and studying. Residents in the NTC-RD group continued their regular schedule of clinical assignments (or routine duties) in the operating rooms. Coping strategies, stressors, social support, anxiety, depression, somatic complaints, and the frequency of alcohol and tobacco use were measured prior to and after the completion of the study.

## Results

Residents in the wellness group reported lower parent role stressors (p=0.03) compared to those in the NTC-RD group, increased social support from work compared to both the NTC-RT group (p =0.02) and NTC-RD group (p = 0.02), decreased anxiety compared to the NTC-RD group (p=0.02), and reduced alcohol consumption compared to those in the NTC-RT group (p=0.02). Residents in the wellness intervention group and the NTC-RT group reported greater increases in problem-solving coping compared to the NTC-RD group (p =0.02 and p = 0.01, respectively).

## Conclusion

Residents in the wellness program reported significantly fewer stressors in their role as a parent, higher levels of perceived social support at work, greater use of problem-solving coping, greater reductions in anxiety, and reduced alcohol consumption.

